# An Efflux Pumps Inhibitor Significantly Improved the Antibacterial Activity of Botanicals from *Plectranthus glandulosus* towards MDR Phenotypes

**DOI:** 10.1155/2021/5597524

**Published:** 2021-05-11

**Authors:** Gravalain Nanmeni, Alex T. Tedonkeu, Aimé G. Fankam, Armelle T. Mbaveng, Brice E. N. Wamba, Paul Nayim, Gabin T. M. Bitchagno, Raïssa T. Nzogong, Maurice D. Awouafack, Mathieu Tene, Veronique P. Beng, Victor Kuete

**Affiliations:** ^1^Department of Biochemistry, Faculty of Science, University of Dschang, Dschang, Cameroon; ^2^Department of Biochemistry, Faculty of Science, University of Yaoundé I, Yaoundé, Cameroon; ^3^Department of Chemistry, Faculty of Science, University of Dschang, Dschang, Cameroon

## Abstract

Bacterial multidrug resistance causes many therapeutic failures, making it more difficult to fight against bacterial diseases. This study aimed to investigate the antibacterial activity of extract, fractions, and phytochemicals from *Plectranthus glandulosus* (Lamiaceae) against multidrug-resistant (MDR) Gram-negative phenotypes expressing efflux pumps. The crude extract after extraction was subjected to column chromatography, and the structures of the isolated compounds were determined using spectrometric and spectroscopic techniques. Antibacterial assays of samples alone and in the presence of an efflux pump inhibitor (phenylalanine-arginine *β*-naphthylamide, PA*β*N) were carried out using the broth microdilution method. The phytochemical study of *P. glandulosus* plant extract afforded seven major fractions (A–G) which lead to the isolation of seventeen known compounds. The ethanol extract of *P. glandulosus* was not active at up to 1024 *μ*g/mL, whereas its fractions showed MICs varying from 32 to 512 *μ*g/mL on the studied bacteria. Fraction C of *P. glandulosus* showed the lowest MIC (32 *μ*g/mL) on *E. coli* ATCC8739 strain. Fraction D presented the highest activity spectrum by inhibiting the growth of 90% (9/10) of the studied bacteria. The presence of PA*β*N has improved the activity of extract and all fractions. Overall, the tested phytochemicals showed low activity against the studied bacteria. The overall results obtained in this study show that some fractions from *P. glandulosus*, mainly fractions C and D, should be investigated more for their possible use to fight against MDR bacteria.

## 1. Introduction

Among infectious diseases, those due to pathogenic bacteria are becoming more worrying. According to the WHO, they are responsible for more than 560,000 deaths or about a fifth of the 2.7 million neonatal deaths per year [1]. Antibiotics, also known as antimicrobial drugs, are drugs used for treating infections caused by bacteria. They have transformed medicine and saved millions of lives [2–4]. Therefore, misuse and overuse of these drugs have contributed to a phenomenon known as antibiotic resistance, which is developed when potentially harmful bacteria change in a way that reduces or eliminates the effectiveness of antibiotics [5, 6]. The consequences of that phenomenon include an increase of hospital stay, the cost of care, as well as the mortality rate [5]. The multidrug resistance observed in Gram-negative bacteria is mainly attributed to the overexpression of efflux pumps via resistance-nodulation cell division (RND) pumps [7]. The fact that bacterial infections can no longer be treated with antibiotics depicts an unknown future in healthcare [8]. To respond to the current challenges of discovering novel antibiotics, it is important to direct research towards new substances. In accordance with this, previous studies have shown that plants are sources of antimicrobial substances and therefore could be as an alternative to fight against infections due to MDR bacteria [4, 9–16].

*Plectranthus glandulosus* Hook. F. (Lamiaceae) is an evergreen perennial flowering herbal plant, highly branched herb up to 3 meters tall, widely distributed in West, Central, and South Africa [17, 18]. It is used in Cameroon's traditional medicine to treat dermatitis, bellyache, venereal diseases, internal inflammation, lower abdominal, and nerve ache [17, 19]. Known as Ava in “Ewondo,” a local tongue in Cameroon, the plant is used as a spice in this part of the country [17]. Some compounds including one methoxylated flavonoid derivative, plectranmicin, and one monoterpene derivative, plectranmicinol, together with seven known compounds including 5-hydroxy-3,7,2′,4′tetramethoxyflavone; 5,7-dihydroxy-3,2′,4′-trimethoxyflavone; 7-hydroxy-5,6,4′-trimethoxyflavone; 3-epi-betulinic acid; 3-*O*-*β*-D-glucopyranosyl; stigmasterol; *β*-sitosterol, and 4-epi-fridelin were isolated from the whole plant of the studies species [20]. Earlier biological studies reported the antinociceptive and anti-inflammatory effects [21] as well as the antioxidant and insecticidal activities of *P. glandulosus* [22, 23].

To contribute to the search for new antibacterial substances, we set out, in this study, to investigate the antibacterial activity of extract, fractions, and isolated compounds from *Plectranthus glandulosus* (Lamiaceae) against MDR Gram-negative phenotypes expressing efflux pumps.

## 2. Materials and Methods

### 2.1. Plant Collection and Extraction

The whole plant of *Plectranthus glandulosus* was collected in Dschang, West Region of Cameroon, in January 2017. Authentication was performed by Mr. Fulbert Tadjouteu, a botanist of the Cameroon National Herbarium in Yaoundé, where our sample was deposited under the voucher number 49084/HNC. The dried and powdered plant material (3.5 kg) was extracted three times (72 h each time) by maceration in EtOH (15 L) at room temperature to afford a crude extract (234 g, 6.7% yield) after filtration, and removal of the solvent was carried out using a rotary evaporator. A portion (224.0 g) of this extract was subjected to silica gel column chromatography (CC) eluting with a mixture of petroleum ether/ethyl acetate (EtOAc) followed by EtOAc/MeOH in increasing polarity. 125 fractions of 400 mL each were collected and combined into six major fractions based on their TLC profiles to afford seven major fractions (A–G).

### 2.2. General Experimental Procedures

MS data were measured on the JEOL MS Station JMS-700 spectrometer or JEOL 600 MS Route spectrometer. High-resolution mass spectra were recorded on a Micromass-Q-TOF-Ultima-3-mass spectrometer (Waters) with lockspray interface and a suitable external calibrant. NMR spectra were recorded on a Bruker Avance III (^1^H-NMR: 600 MHz and ^13^C-NMR: 151.1 MHz), JEOL spectrometers (^1^H-NMR: 500 MHz and ^13^C-NMR 125 MHz), or a Bruker Avance AV-400spectrometer (^1^H-NMR: 400 MHz and ^13^C-NMR 100 MHz). The chemical shifts were reported in parts per million (ppm) with TMS as internal standard. Deuterated solvents, methanol (CD_3_OD), dimethyl sulfoxide (DMSO-d_6_), pyridine (C_5_D_5_N), and chloroform (CDCl_3_) were used as solvents for the NMR experiments. CC was performed with silica gel 60 F254 (70–230 mesh; Merck) and gel permeation with Sephadex LH-20 gel. TLC was carried out with precoated silica gel Kieselgel 60 F254 plates (0.25 mm thick), and spots were detected with UV light (254 and 366 nm) and further sprayed with 20% H_2_SO_4_ reagent followed by heating at 100°C.

### 2.3. Isolation of Constituents

Fraction A (27.0 g) contained mostly fatty material and was not further investigated. Fraction B (46.0 g) was subjected to CC over silica gel eluted with petroleum ether/EtOAc in increasing polarity to give a mixture of *β*-sitosterol and stigmasterol (1 and 2, 30.0 mg). Fraction C (22.0 g) was also subjected to CC over silica gel and eluted with a gradient of *n*-hexane/EtOAc followed by EtOAc/MeOH. 77 fractions of 100 mL each were collected and combined based on their analytical TLC profiles into three main subfractions tagged C1–C3. Subfraction C1 crystallized in hexane/EtOAc (6 : 4) to yield oleanolic acid (3, 3.5 mg). Subfraction C3 was further purified on silica gel eluting with *n*-hexane/EtOAc of increasing polarity to afford pilloin (4, 5.0 mg). Fraction D (18.0 g) was subjected to silica gel CC eluting with a gradient of petroleum ether/EtOAc. 110 fractions of 75 mL each were collected and grouped based on their analytical TLC analysis into 5 subfractions (D1–D5). Subfraction D1 was filtered and washed with EtOAc to give chrysoeriol (6, 18.0 mg). D2 was further subjected to silica gel CC using a gradient of petroleum ether/EtOAc to afford luteolin-7-methyl ether (7, 8.0 mg). D3 was subjected to silica gel CC eluting with the isocratic system *n*-hexane-EtOAc (85 : 15) to afford 5-hydroxy-7,4′-dimethoxyflavone (8, 5.5 mg). The purification of subfraction D5 using isocratic system petroleum ether/EtOAc (1 : 1) afforded a mixture of maslinic acid and benthamic acid (9 + 10, 6.0 mg). D4 was subjected to silica gel CC eluted with a gradient of *n*-hexane/EtOAc to give 5,6-dihydroxy-7,3′,4′-trimethoxyflavone (11, 26.0 mg) and ladanein (12, 31.0 mg). Similarly, elution of fraction *E* (14.5 g) using *n*-hexane/EtOAc followed by EtOAc/MeOH of increasing polarities yielded four subfractions indexed from E1 to E4. E2 was subjected to silica gel CC eluted with a gradient of *n*-hexane/EtOAc to afford three further subfractions (E2-1 to E2-3). The E2-3 subfraction crystallized to give hederagenin (13, 5.5 mg). Filtration of E3, washed with MeOH yielded cylicodiscic acid (14, 4.5 mg). Silica gel CC of fraction F (11.5 g) using CH_2_Cl_2_–MeOH in increasing polarities afforded sitosterol 3-*O*-*β*-D-glucopyranoside (5, 7.5 mg). Fraction G (23.5 g) was also subjected to silica gel CC eluted with a gradient of EtOAc/MeOH to afford six subfractions G1–G6. G1 crystallized at room temperature to afford a mixture of chrysoeriol 5-*O*-*β*-D-glucopyranoside and luteolin-7-*O*-methyl-5-*O*-*β*-D-glucopyranoside (15 + 16, 22.5 mg). Similarly, subfraction G2 crystallized to afford galuteolin (17, 20.5 mg).

### 2.4. Antibacterial Assay

#### 2.4.1. Chemicals

The pure antibiotic chloramphenicol (CHL) ≥ 98% was used as reference antibacterial (RA). *p*-Iodonitrotetrazolium chloride (INT) 0.2% and phenylalanine-arginine*β*-naphthylamide (PA*β*N) ≥ 97% were used as microbial growth indicator and efflux pump inhibitor (EPI), respectively. All these chemicals were provided from Sigma-Aldrich, St. Quentin Fallavier, France. Dimethylsulfoxide (DMSO) 2.5% at the final concentration was used to dissolve the tested samples.

#### 2.4.2. Bacteria Strains and Culture Media and Growth Conditions

A panel of 10 strains belonging to Gram-negative bacteria was used in the study. They included MDR isolates (laboratory collection) and reference strains of *Escherichia coli* (ATCC8739 and AG102), *Enterobacter aerogenes* (ATCC13048 and EA27), *Klebsiella pneumoniae* (ATCC11296 and KP55), *Providencia stuartii* (ATCC 29916 and PS2636), and *Pseudomonas aeruginosa* (PA01 and PA124). The clinical strains were obtained from the laboratory collection from UMR-MD1, University of Marseille, France. The bacterial features are reported in Table S1 (Supplementary Material S1). The bacteria were maintained at 4°C and subcultured overnight on a fresh Mueller–Hinton agar (MHA) before any antibacterial assay.

#### 2.4.3. Bacterial Susceptibility Determination

The antibacterial activity of the extract, fractions, and isolated compounds was determined using INT colorimetric assay [24] with some modifications as previously described [25]. Briefly, samples were dissolved in 10% dimethyl sulfoxide (DMSO)/Mueller–Hinton Broth (MHB) and serially diluted two-fold (in a 96-well microplate). Then, 100 µL of inoculum (2 × 10^6^ CFU/mL) prepared in MHB was added in each well. Chloramphenicol was used as RA, and the well containing the vehicle (DMSO 2.5%) was used as control. The plates were then covered with a sterile plate sealer and gently shaken to mix the contents of the wells. The microplates were incubated at 37°C for 18 h. The minimal bactericidal concentration (MIC) of each sample, defined as the lowest sample concentration that inhibited complete bacteria growth, was detected following the addition of 40 *μ*L INT (0.2 mg/mL) and incubation at 37°C for 30 min. For the minimal bactericidal concentrations (MBCs) determination, a volume of 150 *μ*L of MHB was introduced in a new 96-well microplate, following addition of 50 *μ*L of the previous well microplate contents where no microbial growth was observed and which did not receive an INT (during the reading of MICs). After 48 h incubation at 37°C, the MBC of each sample was determined and defined by adding 40 *μ*L of 0.2 mg/mL INT as described above. Crude extract as well as the active fractions (C and D) were also tested alone and then in the presence of PA*β*N, an efflux pump inhibitor (EPI), at 30 *μ*g/mL final concentration. In this last case, the activity modulation factors (AMFs) were determined by a ratio of MIC sample alone/MIC sample + PA*β*N combination [16]. Each treatment experiment was performed in triplicate, and the assay was repeated thrice.

## 3. Results

### 3.1. Phytochemistry

**Figure 1:**
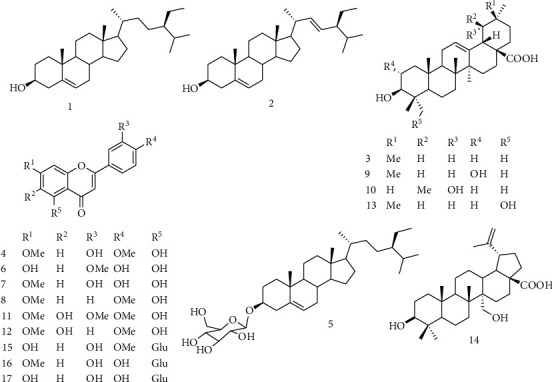
Structures of isolated compounds 1–17.

The EtOH extract of *P. glandulosus* was investigated over silica gel and Sephadex LH-20 column chromatography leading to seventeen compounds. Their structures were determined by spectroscopic techniques and comparison of recorded data with those of similar compounds in the literature (Figure 1). They included the mixture of stigmasterol and *β*-sitosterol (1 + 2) [26], oleanolic acid (3) [27], pilloin (4) [28], *β*-sitosterol 3-*O*-*β*-D-glucopyranoside (5) [29], chrysoeriol (6) [30], luteolin 7-methyl ether (7) [31], 5-hydroxy-7,4′-dimethoxyflavone (8) [32], mixture of maslinic acid (9) [33] and benthamic acid (10) [34], 5,6-dihydroxy-7,3′,4′-trimethoxyflavone (11) [35], ladanein (12) [36], hederagenin (13) [37], cylicodiscic acid (14) [38], mixture of chrysoeriol 5-*O*-*β*-D-glucopyranoside (15) [39] and 7-*O*-methyl luteolin 5-*O*-*β*-D-glucopyranoside (16) [40], and galuteolin (17) [41]. All ^1^H and ^13^C-NMR spectra and major chemical shifts of these compounds are listed in the Supplementary Materials (S2).

### 3.2. Antibacterial Activity of Extract, Fractions, and Compounds

The antibacterial activity of extract, fractions and isolated compounds, and CHL was evaluated against 10 Gram-negative bacteria including reference strains and multidrug-resistant clinical isolates. Tables 1–3 present all these results. Bactericidal or bacteriostatic effects of each sample on a bacterial strain were shown by calculating the MBC/MIC ratio. The EtOH extract of *P. glandulosus* was not active at up to 1024 *μ*g/mL, whereas its fractions showed MICs varying from 32 to 512 *μ*g/mL on the studied bacteria. Fraction C of *P. glandulosus* showed the lowest MIC (32 *μ*g/mL) on *E. coli* ATCC8739 strain. Fraction D presented the highest activity spectrum by inhibiting the growth of 90% (9/10) of the studied bacteria, whereas fractions F–H were not active on the tested bacteria at up to 512 *μ*g/mL. The study showed that all the isolated compounds were generally less active than their main fractions (Table 2) with MICs ranging from 64 to 512 *μ*g/mL. The most active compound, chrysoeriol (6) isolated from the most active fraction, fraction D, showed MICs ranging from 64 to 256 *μ*g/mL against 30% (3/10) of strains (*E. coli* AG102, *K. pneumoniae* ATCC11296, and *P. stuartii* PS2636). Chloramphenicol was active against 100% (10/10) of bacteria with MICs ranging from 2 to 64 *μ*g/mL (Table 1). Except with fraction F, which displayed MBC = 512 *μ*g/mL against *E. aerogenes* EA 27, the other tested plant samples showed no MBC up to 512 *μ*g/mL. However, MBC/MIC ratio of chloramphenicol showed that it has bactericidal effects on most of the tested bacteria (Tables 1 and 2). Fraction F also presented the bactericidal effect agains*t E. aerogenes* EA 27 (MBC/MIC ≤ 4).

### 3.3. Minimal Inhibitory Concentrations of Samples in Association with PA*β*N

Fractions C and D, which showed the best antibacterial activity, the EtOH crude extract, and RA were tested in the presence of PA*β*N, an efflux pumps inhibitor. The overall results (Table 3) showed that, once associated with PA*β*N (30 *μ*g/mL), the activity of all tested samples increased by almost 90% of studied bacteria with activity modulation factors (AMFs) ranging from 2 to 128 for crude extract and fractions and from 4 to 8 for the reference antibiotic, chloramphenicol. PA*β*N mostly potentiated the activity of extract and fraction C (10/10, 100%) than fraction D and CHL (Table 3). The enhancement of the activity was also well observed on *P. aeruginosa* PA124, a known multidrug-resistant bacterium with high expression level of RND type efflux pumps.

## 4. Discussion

Antimicrobial agents are essential in reducing the global burden of infectious diseases. Plants as an inexhaustible source of novel molecules have long been used in traditional medicine for the treatment of several diseases including bacterial infections. Moreover, many techniques for isolation, characterization, and pharmacological evaluation have led to an interest in plant secondary metabolites as a source of new drugs, which are potential antimicrobials [42–44]. Previous chemical studies of that plant resulted in the isolation of many compounds among which those belonging to the phytochemical classes such as flavonoids are known for their antimicrobial activities [20, 42]. In this study, many other known phytochemical compounds have been isolated from *P. glandulosus* (Figure 1), and their antibacterial activities were evaluated.

According to the cutoff values indicating the antibacterial activity of an edible plant extract, its part or its fractions as proposed by Tamokou et al. [45], fractions B–E of *P. glandulosus*, and mainly fractions C and D presented significant activity (100 < MIC ≤ 512 *μ*g/mL) against the tested bacteria strains. Fraction D has the highest activity with MICs ranging from 64 to 512 *μ*g/mL against 90% (9/10) of the studied bacteria (Table 1). Antibiotics and isolated compounds with MIC ≤ 10 *μ*g/mL, 10 < MIC ≤ 100 *μ*g/mL, or MIC > 100 *μ*g/mL are considered to have strong, moderate, or weak activity, respectively [46]. Based on this scale, it is noticed that isolated compounds generally displayed low activity against the tested bacteria (MIC > 100 *μ*g/mL). By comparing the activity of the extract and fractions (Table 1), crude extract (MIC > 1024 *μ*g/mL) was less active than their derived fractions mainly, fractions C and D (MICs ranging from 64 to 256 *μ*g/mL). This suggests that the extract may contain some phytochemicals with antagonistic effects which have been separated by fractionation. Furthermore, in comparing the antibacterial activities of the active fractions (fractions C and D) with that of their respective isolated constituents, it appears that these fractions were more active than the isolated compounds. This insinuates that compounds in each fraction may act synergistically [42, 47]. To the best of our knowledge, this work investigates for the first time antibacterial activity of the extract of *P. glandulosus*. Therefore, Ngassoum et al. [48] revealed low activity of the essential oils of the leaves of *P. glandulosus* against *E. coli*, *P. fluorescens*, and *S. aureus*. This study shows that fractions of *P. glandulosus* could be used to fight infections involving MDR bacteria. Chrysoeriol (6), isolated in *P. glandulosus*, is a methoxylated flavone known for its great scientific interest because of its promising antimicrobial activities against various Gram-negative and Gram-positive bacteria [49]. Its presence in the fraction D may explain the significant antibacterial activity of that fraction.

The activity of tested extract, fractions, as well as chloramphenicol significantly increased in association with PA*β*N, an efflux pump inhibitor (Table 3). In fact, efflux pumps can be blocked by an efflux pump inhibitor, thereby restoring not only the intracellular concentration but also the activity of antibiotics and/or extracts or phytochemicals [50]. This justifies the role of PA*β*N which inhibits or blocks efflux pumps maintaining high intracellular concentrations of antibacterial substances [50, 51]. In the present work, the activity observed in the presence of PA*β*N may indicate that the active principle (*s*) contained in the tested extracts and/or fractions may have been expelled in its absence. This inhibitor would therefore block the efflux pumps and cause an increase in the intracellular concentration of the active principle (*s*) contained in the extracts. This activity therefore confirms that efflux is one of the main resistance mechanisms expressed by the bacteria tested as indicated in Table S1 (Supplementary Materials, SM1).

## 5. Conclusion

The results obtained herein indicate that fractions C and D of *Plectranthus glandulosus* have significant antibacterial activity. Furthermore, this work has shown that the activity of these fractions as well as that of the crude extract can be significantly enhanced in the presence of PA*β*N, an efflux pump inhibitor to overcome MDR bacteria expressing efflux pumps.

## Figures and Tables

**Table 1:** Minimum inhibitory concentration (MIC) and minimum bactericidal concentration (MBC) in *μ*g/mL of crude extracts and fractions from *P. glandulosus* and chloramphenicol against selected Gram-negative bacteria.

Bacteria	Tested samples, MICs (MBCs) in *μ*g/mL								
	Extract	Fraction B	Fraction C	Fraction D	*F*raction E	Fraction F	Fraction G	Fraction H	Chloramphenicol
*Escherichia coli*									
ATCC 8739	-	512 (>512)	32 (>512)	64 (>512)	>512	>512	>512	>512	2 (64)
AG 102	-	512 (>512)	256 (>512)	256 (>512)	512 (>512)	>512)	>512	>512	32 (256)
*Enterobacter aerogenes*									
ATCC 13048	-	512 (>512)	128 (>512)	512 (>512)	512 (>512)	>512	>512	>512	32 (128)
EA 27	-	>512	256 (>512)	512 (>512)	>512	512 (512)	>512	>512	16 (256)
*Klebsiella pneumoniae*									
ATCC 11296	-	512 (>512)	256 (>512)	512 (>512)	512 (>512)	>512	>512	>512	8 (256)
KP 55	-	>512	64 (>512)	512 (>512)	512 (>512)	>512	>512	>512	32 (256)
*Providencia stuartii*									
ATCC 29916	-	>512	>512	512 (>512)	512 (>512)	512 (>512)	>512	>512	64 (256)
PS 2636	-	256 (>512)	256 (>512)	512 (>512)	512 (>512)	>512	>512	>512	64 (256)
*Pseudomonas aeruginosa*									
PA 01	-	256 (>512)	>512	>512	512 (>512)	>512	>512	>512	64 (>256)
PA 124	-	256 (>512)	256 (>512)	512 (>512)	512 (>512)	>512	>512	>512	64 (>256)

(-): ≥1024 *μ*g/mL; MIC, minimum inhibitory concentration; MBC, minimum bactericidal concentration.

**Table 2:** Minimum inhibitory concentration (MIC) and minimum bactericidal concentration (MBC) in *μ*g/mL of isolated compounds of *P. glandulosus* against selected Gram-negative bacteria.

Bacteria	Compounds, MICs (MBCs) in *μ*g/mL				
	6	11	12	15 + 16	17
*Escherichia coli*					
ATCC 8739	-	-	-	-	-
AG 102	128 (-)	-	-	64 (-)	-
*Enterobacter aerogenes*					
ATCC 13048	-	-	-	-	-
EA 27	-	-	-	-	-
*Klebsiella pneumoniae*					
ATCC 11296	256 (-)	64 (-)	-	64 (-)	64 (-)
KP 55	-	-	-	-	-
*Providencia stuartii*					
ATCC 29916	-	-	-	256 (-)	512 (-)
PS 2636	64 (-)	256 (-)	64 (-)	512 (-)	-
*Pseudomonas aeruginosa*					
PA 01	-	-	-	-	-
PA 124	-	-	-	-	-

(-): ≥512 *μ*g/mL; MIC, minimum inhibitory concentration; MBC, minimum bactericidal concentration; 6, chrysoeriol; 11, 5,6-dihydroxy-7,3′,4′-trimethoxyflavone; 12, ladanein; 15, chrysoeriol 5-*O*-*β*-D-glucopyranoside; 16, 7-*O*-methyl luteolin 5-*O*-*β*-D-glucopyranoside; 17, luteolin 5-*O*-*β*-D-glucopyranoside.

**Table 3:** MICs (*μ*g/mL) of extract, active fractions of *P. glandulosus* and chloramphenicol in the presence of PA*β*N.

Bacteria	Tested samples, MIC in the presence of PA*β*N (AMFs) (*μ*g/mL)			
	Extract	Fraction C	Fraction D	Chloramphenicol
*Escherichia coli*				
ATCC 8739	<8 (nd)	<4 (>8)	<4 (>16)	<2 (nd)
AG 102	<8 (nd)	8 (32)	<4 (>64)	8 (4)
*Enterobacter aerogenes*				
ATCC 13048	32 (nd)	64 (2)	256 (2)	32 (1)
EA 27	<8 (nd)	<4 (>64)	32 (16)	16 (1)
*Klebsiella pneumoniae*				
ATCC 11296	<8 (nd)	<4 (>64)	<4 (128)	8 (1)
KP 55	32 (nd)	32 (2)	64 (8)	32 (1)
*Providencia stuartii*				
ATCC 29916	<8 (nd)	64 (4)	256 (2)	8 (8)
PS 2636	512 (nd)	<4 (64)	256 (2)	16 (4)
*Pseudomonas aeruginosa*				
PA 01	<8 (nd)	>512 (nd)	>512 (nd)	8 (8)
PA 124	256 (nd)	64 (4)	512 (1)	16 (4)

MIC, minimum inhibitory concentration; nd, not determined. Activity modulation factors (AMFs) were determined to qualify the potentiation level of sample activity in the presence of EPI. All assays were performed in triplicate and repeated thrice. In bold are AMF ≥ 4.

## Data Availability

All data generated or analyzed during this study are included within this article.

## Supplementary Materials

Supplementary Materials Additional file. Docx: SM1. Table S1 showing the characteristics of the studied bacteria; SM2. ^1^H and ^13^C-NMR and major chemical shifts of studied compounds.
