# Supplementary material for: An Efflux Pumps Inhibitor Significantly Improved the Antibacterial Activity of Botanicals from Plectranthus glandulosus towards MDR Phenotypes
Source: ScientificWorldJournal. 2021 May 11;2021:5597524. doi: 10.1155/2021/5597524 (PMC8131149; doi:10.1155/2021/5597524)
Supplement: Supplementary Materials — Additional file. Docx: SM1. Table S1 showing the characteristics of the studied bacteria; SM2. 1H and 13C-NMR and major chemical shifts of studied compounds. [file 5597524.f1.doc]

# Phenylalanine-arginine *β*-naphthylamide, an efflux pumps inhibitor, significantly improved the antibacterial activity of botanicals from *Plectranthus glandulosus* (Lamiaceae) towards multidrug-resistant Gram-negative phenotypes.

Gravalain Nanmeni^1,2^, Alex T. Tedonkeu^3^, Aimé G. Fankam^1^, Armelle T. Mbaveng^1*^, Brice E.N. Wamba^1^, Paul Nayim^1^, Gabin T.M. Bitchagno^3^, Raïssa T. Nzogong^3^, Maurice D. Awouafack^3^, Mathieu Tene^3^, Veronique P. Beng^2^ and Victor Kuete^1**^

*^1^Department of Biochemistry, Faculty of Science, University of Dschang, Dschang, Cameroon*

*^2^Department of Biochemistry, Faculty of Science, University of Yaoundé I, Yaoundé, Cameroon*

*^3^Department of Chemistry, Faculty of Science, University of Dschang, Dschang, Cameroon*

***Corresponding authors:***

** E-mail:* [*armkuete@yahoo.fr*](mailto:armkuete@yahoo.fr)*; P.O. Box 67 Dschang, Cameroon (Dr Armelle T. Mbaveng)*

***Tel: (+237) 677355927; E-mail:* [*kuetevictor@yahoo.fr*](mailto:kuetevictor@yahoo.fr)*; P.O. Box 1499 Bafoussam, Cameroon (Prof. Dr. Victor Kuete)*

***Other author’s emails***

*Gravalain Nanmeni:* [*nanmenigravalain@yahoo.com*](mailto:nanmenigravalain@yahoo.com)

*Alex T. Tedonkeu:* [*alextedonkeu@yahoo.fr*](mailto:alextedonkeu@yahoo.fr)

*Aimé G. Fankam:* [*agfankam@yahoo.fr*](mailto:agfankam@yahoo.fr)

*Brice E.N. Wamba:* [*wambaelvis@yahoo.fr*](mailto:wambaelvis@yahoo.fr)

*Paul Nayim:* [*nayimpaul@yahoo.fr*](mailto:nayimpaul@yahoo.fr)

*Gabin T.M. Bitchagno:* [*bmgt198716@ymail.com*](mailto:bmgt198716@ymail.com)

*Raïssa T. Nzogong:* [*ntraissa69@yahoo.fr*](mailto:ntraissa69@yahoo.fr)

*Maurice D. Awouafack:* [*amauduc2@yahoo.com*](mailto:amauduc2@yahoo.com)

*Mathieu Tene:* [*mtene2001@yahoo.fr*](mailto:mtene2001@yahoo.fr)

*Veronique P. Beng:* [*v.penlap@yahoo.fr*](mailto:v.penlap@yahoo.fr)

**Supporting material SM 1.** Table S1 showing the characteristics of the studied bacteria

**Table S1. Gram-negative bacteria and their features**

| **Strains** | **Features** | **References** |
| --- | --- | --- |
| ***Escherichia coli*** |  |  |
| ATTC 8739 | Reference strain |  |
| AG 102 | AG 100 expressing *Acr AB* pumps | [ 1] |
| ***Enterobacter aerogenes*** |  |  |
| ATCC 13048 | Reference strain |  |
| EA 27 | Clinical MDR isolate exhibiting energy-dependent norfloxacin and chloramphenicol efflux with KAN^R^ and AMP^R^ and NAL^R^ and STR^R^ and TET^R^ | [2] |
| ***Klebsiella pneumoniae*** |  |  |
| ATCC11296 | Reference strain |  |
| Kp 55 | Clinical MDR isolate, TET^R^, AMP^R^, ATM^R^, and CEF^R^ | [3] |
| ***Providencia stuartii*** |  |  |
| ATCC29916 | Reference strain | [ 4] |
| PS2636 | Clinical MDR isolate, *AcrAB-TolC* |
| ***Pseudomonas aeruginosa*** |  |  |
| PA 01 | Reference strain |  |
| PA 124 | Clinical MDR isolate | [5 ] |

^a^AMP^R^, ATM^R^, CEF^R^, CFT^R^, CHL^R^, CIPR, ERM^R^, FEP^R^, FLX^R^, IM/CS^R^, KAN^R^, MOX^R^, OFX^R^, STR^R^, TET^R^: Resistance to ampicillin, aztreonam, cephalothin, cefadroxil, chloramphenicol, Ciprofloxacin, Erythromycin, cefepime,Flomoxef, Imipenem/ Cilastatinsodium, kanamycin, moxalactam, streptomycin, andtetracycline; MDR : Multidrug-resistant.

**References**

[1] C. A. Elkins, and L. B. Mullis, “Substrate competition studies using whole-cell accumulation assays with the major tripartite multidrug efflux pumps of *Escherichia coli*,” *Antimicrobial Agents and Chemotherapy*, vol. 51, no. 3, pp. 923–929, 2007.

[2] M. Mallea, A. Mahamoud, J. Chevalier, et al., Alkylamino- quinolines inhibit the bacterial antibiotic efflux pump in multidrug-resistant clinical isolates,” *Biochemical Journal*, vol. 376, no. 3, pp. 801–805. 2003.

[3] M. Mallea, J. Chevalier, C. Bornet, et al., “Porin alteration and active efflux: two *in vivo* drug resistance strategies used by *Enterobacter aerogenes*,” *Microbiology*, vol. 144, no. 11, pp. 3003–3009, 1998.

[4] J. Chevalier, J-M. Pages, A. Eyraud, and M. Mallea, “Membrane permeability modifications are involved in antibiotic resistance in *Klebsiella pneumonia*,” *Biochemical and Biophysical Research Communications*, vol. 274, no. 2, pp. 496–499, 2000.

[5] Q-T. Tran, K. R. Mahendra, A. Hajjar, et al., “Implication of porins in ß-lactam resistance of *Providencia stuartii*,” *Journal of Biological Chemistry*, vol. 285, pp. 32273-81, 2010.

[6] V. Lorenzi, A. Muselli, A. F. Bernardini, et al., “Geraniol restores antibiotic activities against multidrug-resistant isolates from Gram-negative species,” *Antimicrobial Agents and Chemotherapy*, vol. 53, pp. 2209–2211, 2009.

**Supporting materials SM2:** ^1^H and ^13^C NMR and major chemical shifts of studied compounds

- Mixture of stigmasterol and *β*-sistosterol (1+2)

Amorphous powder, C_29_H_50_O, *m/z* 414 and C_29_H_48_O, *m/z* 412; ^1^H NMR (CDCl_3_, 400 MHz): 5.35 (d, *J* = 4.9 Hz, H-6), 5.12 (m, H-22), 5.01 (m, H-23), 3.52 (m, H-3), 2.27 (m, H-4), 1.01 (s, H-18), 0.81 (s, H-19). ^13^C NMR (CDCl_3_, 100 MHz): *β*-*sitosterol* 140.7 (C-5), 121.7 (C-6), 71.8 (C-3), 56.7 (C-14), 59.9 (C-17), 50.1 (C-9), 45.8 (C-24), 42.3 (C-4), 42.3 (C-13), 39.7 (C-12). *Stigmasterol:* 140.7 (C-5), 121.7 (C-6), 71.8 (C-3), 56.8 (C-14), 56.0 (C-17), 51.2 (C-24), 50.1 (C-9), 43.3 (C-4), 42.3 (C-13), 39.8 (C-12).

Figure S1: Full^1^H NMR spectrum (CDCl_3_ 400 MHz) of compounds **1+2**

Figure S2: Full ^13^C NMR spectrum (CDCl_3_, 100 MHz) of compounds **1**+**2**

- Oleanolic acid (**3**)

White powder, C_30_H_48_O_3_, *m/z* 456, mp 170
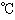
, ^13^C NMR (DMSO, 100 MHz): 182.7 (C-28), 143.6 (C-13), 122.6 (C-12), 79.0 (C-3), 55.2 (C-5), 47.6 (C-9), 46.5 (C-17), 45.9 (C-19), 41.6 (C-14), 41.0 (C-18), 39.2 (C-8), 38.7 (C-4), 38.4 (C-1), 37.0 (C-10), 33.8 (C-21), 33.0 (C-29), 32.6 (C-7), 32.4 (C-22), 30.7 (C-20), 28.1 (C-23), 27.7 (C-15), 27.1 (C-2), 25.9 (C-27), 23.6 (C-30), 23.4 (C-16), 23.0 (C-11), 18.3 (C-6), 17.1 (C-26), 15.5 (C-24), 15.3 (C-25). ^1^H NMR (DMSO, 400 MHz): 5.49 (1H, s, H-12), 3.47 (1H, t, *J* = 8.0 Hz, H-3), 3.30 (1H, m, H-18), 1.60 (2H, m, H-2), 1.52 (2H, m, H-6), 1.12 (3H, s, CH_3_-27), 0.97 (2H, m, H-1), 0.96 (3H, s, CH_3_-30), 0.91 (3H, s, CH_3_-25), 0.89 (3H, s, CH_3_-23), 0.87 (3H, s, CH_3_-24), 0.75 (3H, s, CH_3_-26), 0.70 (1H, m,H-5).


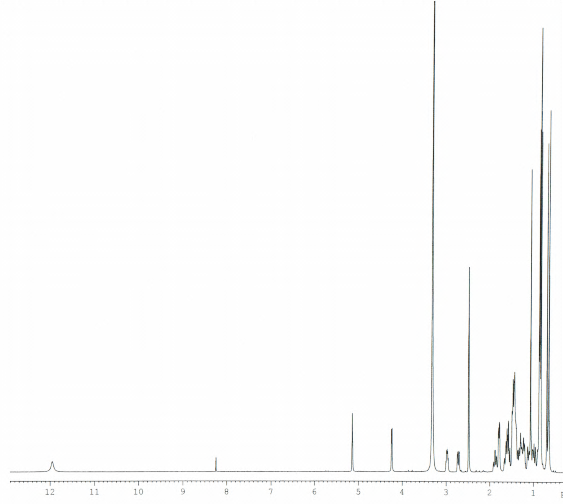


Figure S3: Full ^1^H NMR spectrum (DMSO-*d6*, 400 MHz) of compound **3**


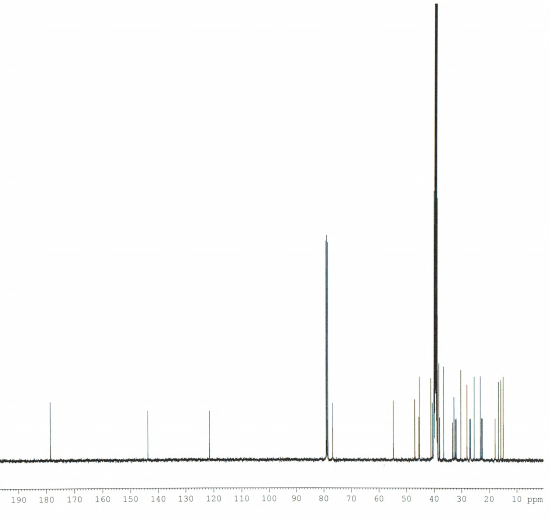


Figure S4: Full ^13^C NMR spectrum (DMSO-*d_6_*, 100 MHz) of compound **3**

- Pilloin **(4)**

Yellow powder, C_17_H_14_O_6_, *m/z* 313; ^1^H NMR (CDCl_3_, 600 MHz):7.56 (d, *J* = 8.5 Hz, H-6’), 6.93 (s, H-3), 6.89 (d ; *J* = 8,5 Hz, H-5’), 6.33 (H-6), 6.77 (s, H-8), 3.83 (7-OMe) ,3.85 (4’-OMe). ^13^C NMR (CDCl_3_, 150 MHz): 183.3 (C-4), 165.5 (C-7), 164.4 (C-2), 161.9 (C-5), 157.7 (C-9), 151.3 (C-4’), 148.5 (C-3’), 103.9 (C-3), 121.8 (C-1’), 121.0 (C-6’), 116.2 (C-5’), 110.6 (C-2’), 105.2 (C-10), 98.2 (C-6), 93.1 (C-8), 56.6 (4’-OMe), 56.5 (7-OMe)


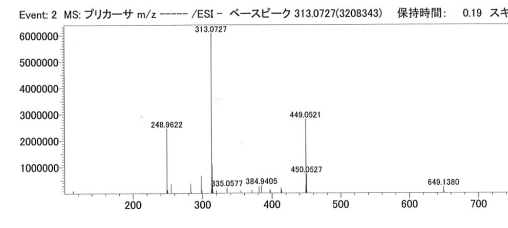


Figure S5: ESI mass of compound **4**

Figure S6: Full ^1^H NMR spectrum (CDCl_3_, 600 MHz) of compound **4**

Figure S7: Full ^13^C NMR spectrum (CDCl_3_, 150 MHz) of compound **4**

- Sitosterol 3-*O*-*β*-D-glucopyranoside (**5**)

Belgian powder, C_35_H_60_O_6_, m/z 576, ^13^C NMR (DMSO-*d6*, 150 MHz): 140.8 (C-5), 121.7 (C-6), 101.2 (C-1’), 77.2 (C-3), 77.2 (C-3’), 76.2 (C-5’), 73.9 (C-2’), 70.5 (C-4’), 61.5 (C-6’) 56.6 (C-14), 55.9 (C-17), 50.0 (C-9), 45.6 (C-24), 43.3 (C-13), 39.4 (C-12), 38.7 (C-4), 37.3 (C-1), 36.9 (C-10), 35.9 (C-20), 33.8 (C-22), 31.8 (C-8), 31.8 (C-7), 29.7 (C-2), 29.1 (C-25), 28.2 (C-16), 25.8 (C-23), 24.3 (C-15), 23.0 (C-28), 21.0 (C-11), 19.5 (C-27), 19.4 (C-26), 19.0 (C-19), 19.0 (C-21), 12.2 (C-18), 12.1 (C-29).


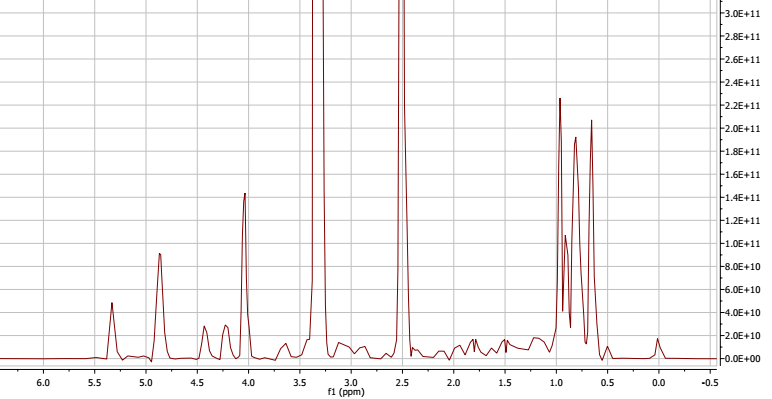


Figure S8: Full ^1^H NMR spectrum (DMSO-*d_6_*, 600 MHz) of compound **5**


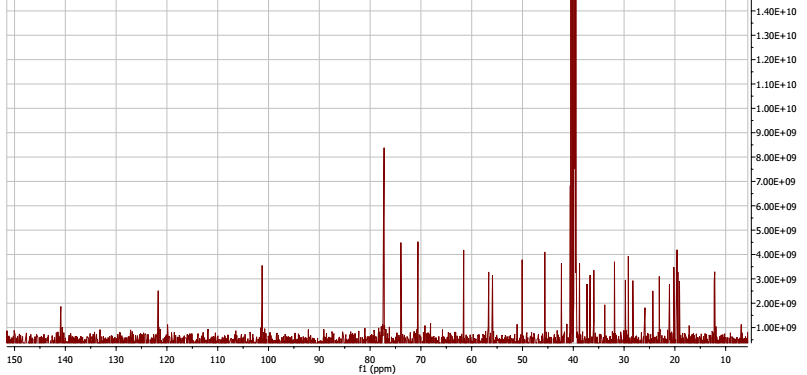


Figure S9: Full ^13^C NMR spectrum (DMSO-*d6*, 150 MHz) compound **5**

- Chrysoeriol (**6**)

**6**

Yellow powder, C_16_H_12_O_6_, *m/z* 300, ^1^H NMR (C_5_D_5_N, 600 MHz), 7.55 (m, H-6’), 7.51 (d. 2.2 Hz, H-2’), 7.18 (d, *J* = 8.8 Hz, H-5’), 6.87 (s, H-3), 6.76 (d, *J* = 2.0 Hz, H-8), 6.66 (d, *J* = 2.0 Hz, H-3), 3,75 (s, 3’-OMe). ^13^C NMR (C_5_D_5_N, 150 MHz): 182.2 (C-4), 165.8 (C-7), 164.8 (C-2), 162.3 (C-5), 158.3 (C-9), 152.8 (C-4’), 148.6 (C-3’), 122.3 (C-1’), 121.2 (C-6’), 116.6 (C-5’), 110.0 (C-2’), 104.8 (C-10), 103.9 (C-3), 99.8 (C-6), 94.6 (C-8), 54.1 (3’-OMe).


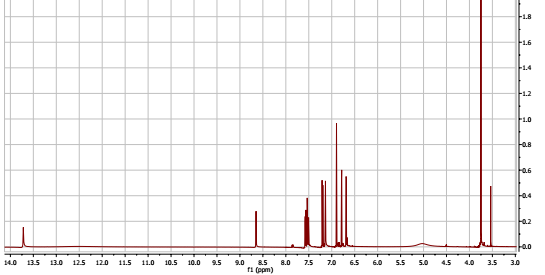


Figure S10: Full ^1^H NMR spectrum (C_5_D_5_N, 600 MHz) of compound **6**


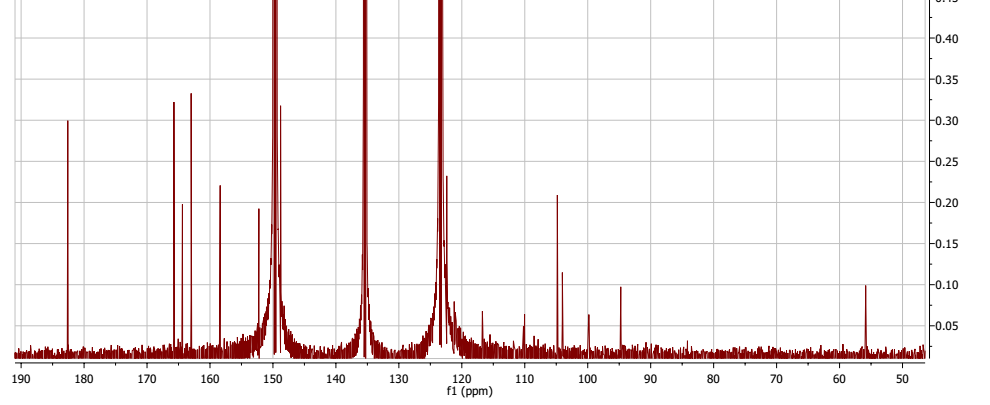


Figure S11: Full ^13^C NMR spectrum (C_5_D_5_N, 150 MHz) of compound **6**

- Luteolin-7-methyl ether (**7**)

**7**

Yellow powder, C_16_H_12_O_6_, *m/z* 300, ^1^H NMR (CDCl_3_, 600 MHz): 7.41 (dd, *J* = 8.4, 2.2 Hz, H-6’), 7.38 (d, *J* = 2.2 Hz, H-2’), 6.95 (d, *J* = 8.5 Hz, H-5’), 6,55 (s, H-3), 6.53 (d, *J* = 2.2 Hz, H-8), 6.37 (d, *J* = 2.2 Hz, H-6), 3.89 (s, 7-OMe). ^13^C NMR (CDCl_3_, 150 MHz): 182.7 (C-4), 165.0 (C-7), 164.8 (C-2), 161.6 (C-9), 103.2 (C-3), 157.8 (C-5), 149.0 (C-4’), 145.3 (C-3’), 122.7 (C-1’), 119.4 (C-6’), 115.1 (C-5’), 112.9 (C-2’), 105.3 (C-10), 98.2 (C-6), 92.6 (C-8), 55.9 (7-OMe).

Figure S12: Full ^1^H NMR spectrum (CDCl_3_, 600 MHz) of compound **7**

Figure S13: Full ^13^C NMR spectrum (CDCl_3_, 150 MHz) of compound **7**

- 5-hydroxy-7-4’ dimethoxyflavone (**8**)

**8**

Whitish powder, C_17_H_14_O_5_, *m/z* 299, , ^1^H NMR (CDCl_3_, 600 MHz): 7.85 (*d*, *J* = 8.5 Hz, H-2’/H-6’), 7.02; (*d*, *J* = 8.5 Hz, H-3’/H-5’), 6.55; (s, H-3), 6.49 (d; *J* = 2.5 Hz, H-6), 6.35 (*d*, *J* = 2.5 Hz, H-8), 3,88 (s, 7-OMe), 3,85 (s, 4’-OMe). ^13^C NMR (CDCl_3_, 150 MHz), 182.5 (C-4), 165.5 (C-7), 164.3 (C-2), 162.2 (C-9), 157.7 (C-5), 128.1 (C-2’/C-6’), 123.7 (C-1’), 114.4 (C3’/C5’), 105.7 (C-10), 104.5 (C-3), 98.1 (C-6), 92.8 (C-8), 55.6 (7-OMe), 55.9 (4’-OMe).


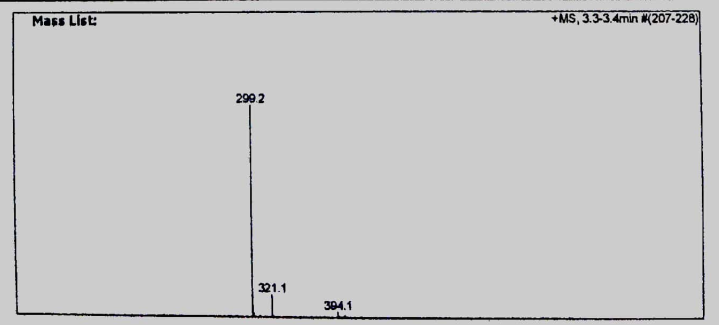


Figure S14: ESI mass spectrum of compound **8**


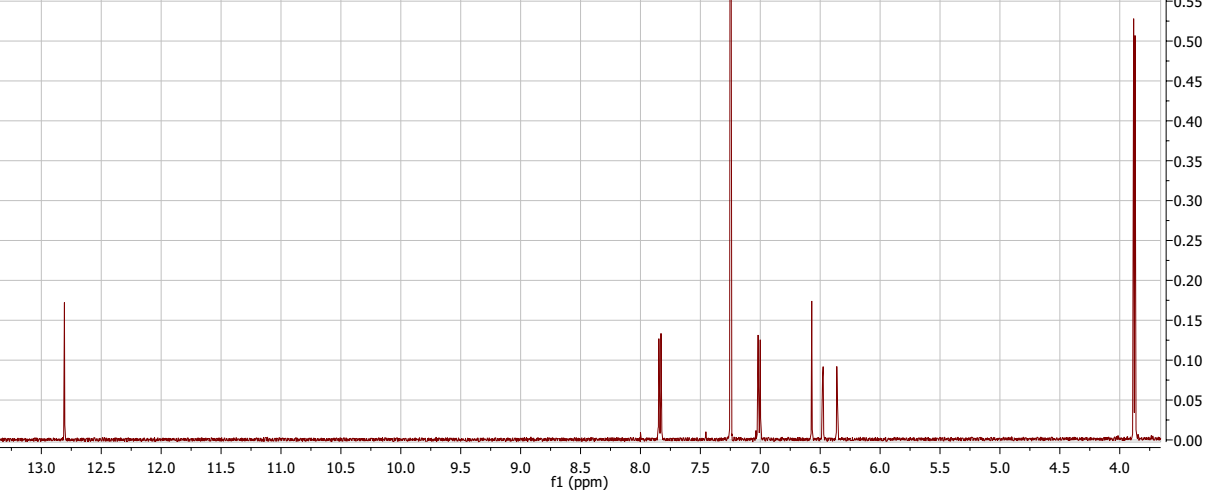


Figure S15: Full ^1^H NMR spectrum (CDCl_3_, 600 MHz) of compound **8**


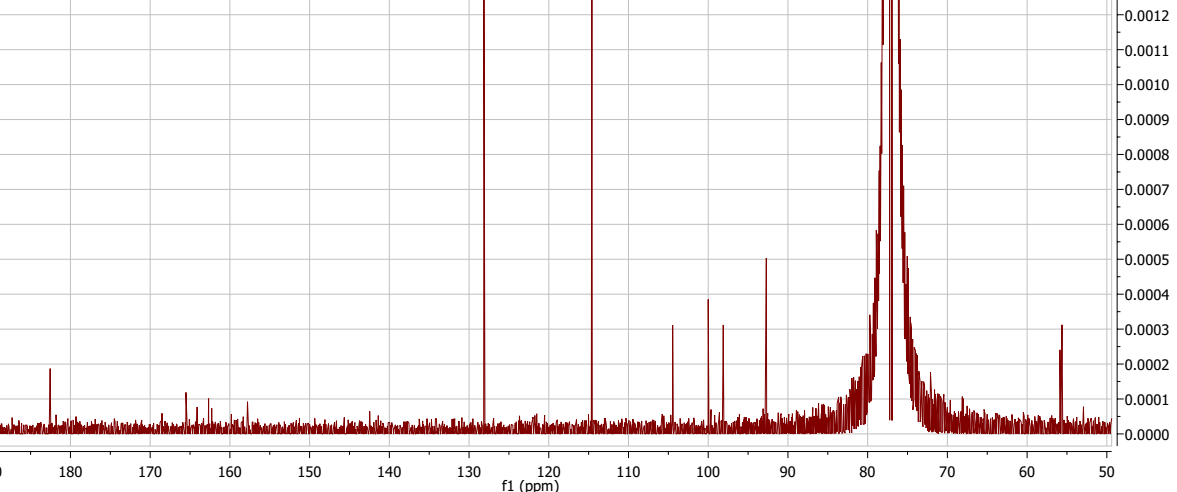


Figure S16: Full ^13^C NMR spectrum (CDCl_3_, 150 MHz) of compound **8**

- Mixture of maslinic acid (**9**) and benthamic acid (**10**)

**9**

White powder, C_30_H_48_O_4_, *m/z* 495, ^13^C NMR (DMSO-*d6*, 150 MHz): maslinic acid: 178.9 (C-28), 144.4 (C-13), 121.9 (C-12), 82.7 (C-3), 67.7 (C-2), 55.2 (C-5), 46.0 (C-1), 47.5 (C-18), 47.5 (C-9), 46.2 (C-17), 45.6 (C-19), 41.7 (C-14), 39.5 (C-4), 39.1 (C-8), 38.2 (C-10), 33.8 (C-21), 33.2 (C-7), 32.8 (C-29), 32.7 (C-22), 30.5 (C-20), 30.0 (C-23), 27.8 (C-15), 25.7 (C-27), 24.1 (C-11), 23.8 (C.16), 23.0 (C-30), 18.2 (C-6), 17.5 (C-26), 17.1 (C-24), 16.4 (C-25). Benthamic acid: 178.9 (C-28), 138.7 (C-13), 121.7 (C-12), 78.4 (C-3), 72.1 (C-19) Mahato et Kundu, 1994


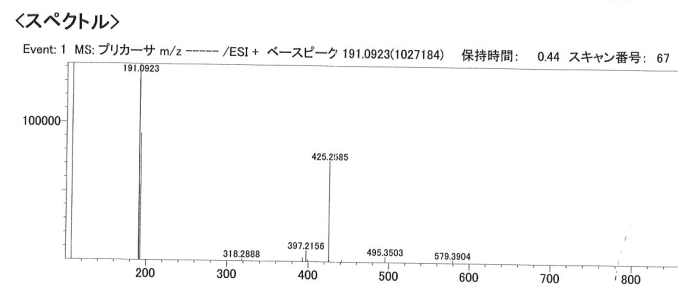


Figure S17: ESI mass of compound **9** + **10**


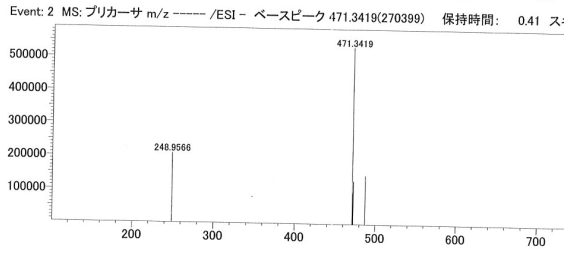


Figure S18: ESI mass Spectrum of compound **9** + **10**

Figure S19: Full ^1^H NMR spectrum (DMSO-*d_6_*, 600 MHz) compounds **9** + **10**

- 5,6-dihydroxy-7,3’,4' trimethoxyflavone (**11**)

**11**

Yellow powder, C_18_H_16_O_7_, *m/z* 367, ^1^H NMR (C_5_D_5_N, 600 MHz): 7.60 (m, H-6’), 7.54 (m, H-2’), 7.01 (m, H-5’), 6.88 (s, H-3), 6.66 (m, H-8), 3.80 (s, 7-OMe), 3.60 (s, 4’-OMe), 3.52 (s, 3’-OMe). ^13^C NMR (C_5_D_5_N, 150 MHz): 182.9 (C-4), 163.7 (C-2), 155.1 (C-7), 152.7 (C-4’), 149.6 (C-3’), 149.6 (C-9), 149.5 (C-5), 124.1 (C-1’), 120.0 (C-6’), 104.1 (C-3), 131.2 (C-6), 111.8 (C-5’), 109.6 (C-2’), 105.4 (C-10), 91.5 (C-8), 56.9 (7-OMe), 55.8 (3’-OMe), 54.7 (4’-OMe).


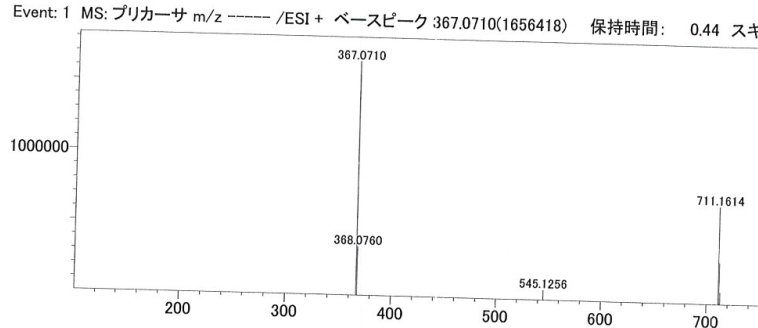


Figure S20: ESI spectrum mass of compound **11**

Figure S21: Full ^1^H NMR spectrum (C_5_D_5_N, 600 MHz) compound **11**

Figure S22: Full ^13^C NMR spectrum (C_5_D_5_N, 150 MHz) compound **11**

- Ladanein (**12**)

**12**

Yellow powder, C_17_H_14_O_6_, *m/z* 337, ^1^H NMR (DMSO-*d_6_*, 600 MHz): 8.01 (d, *J* = 8.9 Hz, H-2’/H-6’), 7.07 (d, *J* = 9 Hz, H-3’/5’), 6.87 (s, H-8), 6.83 (s, H-3), 3.87 (s, 7-OMe), 3.80 (s, 4’-OMe). ^13^C NMR: (DMSO-*d_6_*, 150 MHz): 182.2 (C-4), 163.7 (C-2), 162.7 (C-4’), 155.3 (C-7), 150.4 (C-9), 146.7 (C-5), 130.0 (C-6), 128.2 (C-2’/C-6’), 123.5 (C-1’), 114.5 (C-3’/C-5’), 105.4 (C-10), 103.5 (C-3), 91.1 (C-8), 57.0 (4’-OMe), 56.7 (7-OMe).


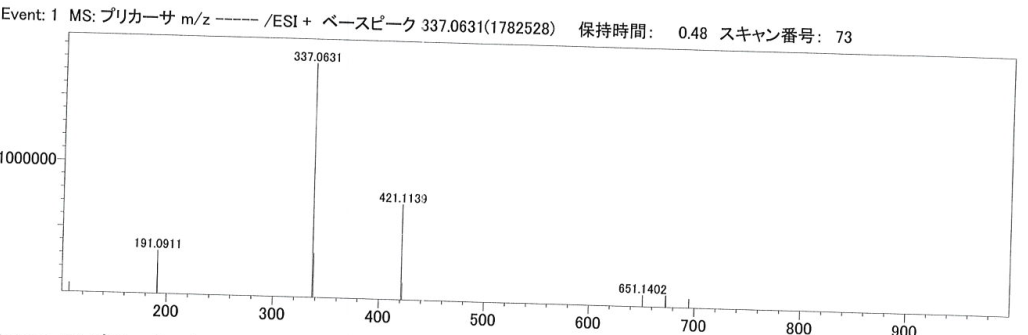


Figure S23: ESI mass spectrum of compound **12**

Figure S24: Full ^1^H NMR spectrum (DMSO-*d6*, 600 MHz) compound **12**

Figure S25: Full ^13^C NMR spectrum (DMSO-*d6*, 150 MHz) compound **12**

- Hederagenin (**13**)

**13**

Greenish powder, C_30_H_48_O_4_, m/z 495, ^1^H NMR (CDCl_3_, 600 MHz): 5.17 (t, *J* = 3.7 Hz, H-12), 3.52 (dd, *J* = 11.3, 4.6 Hz, H-3), 3.49 (m, H-24*α*), 3.25 (m, H-24*β*), 2.74 (dd, *J* = 13.9, 4.6 Hz, H- 18), 1.06 (s, H-27), 0.87 (s, H-26), 0.84 (s, H-30), 0.81 (s, H-29), 0.80 (s, H-25), 0.6 (s, H-23). ^13^C NMR (CDCl_3_, 150 MHz): 180.0 (C-28), 143.2 (C-13), 121.5 (C-12), 73.6 (C-3), 67.8 (C-24), 47.7 (C-5), 46.8 (C-17), 46.8 (C-9), 45.1 (C-19), 41.1 (C-18), 41.0 (C-14), 40.6 (C-4), 38.4 (C-8), 37.4 (C-1), 36.0 (C-21), 36.0 (C-10), 33.0 (C-22), 33.0 (C-7), 31.5 (C-20), 31.5 (C-29), 28.8 (C-15), 26.8 (C-2), 24.8 (C-27), 22.5 (C-11), 22.5 (C-16), 22,4 (C-30).17.4 (C-6), 14.6 (C-26), 12.9 (C-25), 10.7 (C-23).


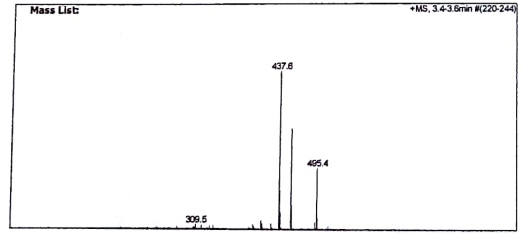


Figure 26: ESI mass spectrum of compound **13**

Figure S27: Full ^1^H NMR spectrum (CDCl_3_, 600 MHz) of compound **13**

Figure S28: Full ^13^C NMR spectrum (CDCl_3_, 150 MHz) compound **13**

- Cylicodiscic acid (**14**)

**14**

White power, C_30_H_48_O_4_, 472, ^1^H NMR (CD_3_OD, 600 MHz): 4.73 (d, *J* = 2.3 Hz, H-29α), 4.61 (dd, *J* = 2.4, 1.4 Hz, H-29*β*), 3,76 (d; *J* =10,5 Hz, H-27 *β*), 4,18 (d, *J* =10,5 Hz, H-27*α*), 3.16 (dd, *J* = 11.6, 4.7 Hz, H-3), 3.04 (td, *J* = 10.8, 5.0 Hz, H-19), 2.41 (ddd, *J* = 13.5, 11.6, 3.5 Hz, H-13), 1,71 (s, H-30), 0,99 (s, H-26), 0.90 (s, H-25), 0,93 (s, H-23), 0,81 (m, H-5), 0,77 (s, H-24). ^13^C NMR (CD_3_OD, 150 MHz): 178.8 (C-28), 151.1 (C-20), 109.2 (C-29), 78.2 (C-3), 59.5 (C-27), 55.8 (C-17), 55.5 (C-5), 51.8 (C-9), 49.3 (C-18), 47.2 (C-19), 46.1 (C-14), 41.1 (C-8), 38.9 (C-1), 38.8 (C-13), 38.5 (C-4), 37.0 (C-22), 37.0 (C-10), 35.1 (C-7), 32.7 (C-16), 30.2 (C-21), 27.1 (C-23), 26.5 (C-2), 24.6 (C-12), 23.0 (C-15), 20.6 (C-11), 18.3 (C-30) 18.1 (C-6), 14.7 (C-24), 15.7 (C-25), 15.6 (C-26).

Figure S29: Full ^1^H NMR spectrum (CDOD_3_, 600 MHz) of compound **14**

Figure S30: Full ^13^C NMR spectrum (CDOD_3_, 150 MHz) compound **14**

- Mixture of Chrysoeriol 5-*β*-D-glucopyranoside (**15**) and Luteolin 7-O-methyl-5-O- glucopyranoside (**16**)

**16**

Yellowish; C_22_H_22_O_11_, m/z 462, ^1^H NMR (DMSO-*d_6_*, 600 MHz): Chrysoeriol 5-*β*-D-glucopyranoside: 7.48 (m, H-6’), 7.47 (d, *J* = 2.6 Hz, H-2’), 6.76 (d, *J* = 2.7 Hz, H-6), 6.89 (d, *J* = 8.8 Hz, H-5’), 6.65 (d, *J* = 2.7 Hz, H-8), 6,50 (s H-3), 3.84 (s, 3’-OMe). Luteolin 7-O-methyl-5-O- glucopyranoside: 7.34 (d, *J* = 2.3 Hz, H-6’), 7.32 (d, *J* = 2.6 Hz, H-2’), 7.05 (d, *J* = 2.7 Hz, H-8), 6.87 (d, *J* = 2.7 Hz, H-6), 6.85 (d, *J* = 8.8 Hz, H-5’), 6.72 (s, H-3), 3.86 (s, 7-OMe). ^13^C NMR (DMSO-*d_6_*, 150 MHz): ^13^C NMR (DMSO-*d6*, 150 MHz): 177.5 (C-4), 163.2 (C-7), 161.7 (C-2) , 159.1 (C-5), 158.9 (C-9), 150.9 (C-4’), 148.8 (C-3’), 122.0 (C-1’), 120.4 (C-6’), 116.3 (C-5’), 110.7 (C-2’), 108.7 (C-10), 106.2 (C-3), 105.1 (C-1’’), 104.8 (C-6), 98.9 (C-8), 78.1 (C-3’’), 76.1 (C-5’’), 74.2 (C-2’’), 70.2 (C-4’’), 61.4 (C-6’’), 56.4 (3’-OMe). Luteolin 7-O-methyl-5-O- glucopyranoside: 177.7 (C-4), 164.0 (C-7), 161.7 (C-2), 158.5 (C-5), 158.8 (C-9), 149.8 (C-4’), 146.3 (C-3’), 122.0 (C-1’), 119.0 (C-6’), 116.2 (C-5’), 113.6 (C-2’), 109.1 (C-10), 106.5 (C-3), 104.5 (C-1’’), 104.0 (C-6), 97.1 (C-8), 78.1 (C-3’’), 76.1 (C-5’’), 74.2 (C-2’’), 70.2 (C-4’’), 61.4 (C-6’’), 56.4, (7-OMe).

Figure S31: Full ^1^H NMR spectrum (DMSO-*d_6_*, 600 MHz) of compound **15** + **16**

Figure S32: Full ^13^C NMR spectrum (DMSO-*d_6_*, 150 MHz) compound **15** + **16**

- Galuteolin (**17**)

Yellow powder, C_21_H_20_O_11_, *m/z* 449, ^1^H NMR (DMSO-*d_6_*, 600 MHz). 7.34 (dd, *J* = 2.5, 12 Hz, H-6’), 7.31 (*sl,* H-2’), 6.50 (s, H-3), 6.75 (d, *J* = 3 Hz, H-6), 6.85 (*d*, *J* = 10.5 Hz, H-5’), 6.65 (d, *J* = 3 Hz, H-8), 4.64 (*d*, *J* = 9 Hz, H-1’’). ^13^C NMR (DMSO-*d_6_*, 150 MHz): 177.4 (C-4), 163.0 (C-7), 161.8 (C-2), 159.0 (C-5), 158.8 (C-9), 149.5 (C-4’), 146.0 (C-3’), 122.1 (C-1’), 119.0 (C-6’), 116.6 (C-5’), 113.6 (C-2’), 108.6 (C-10), 106.1 (C-3), 105.0 (C-1’’), 104.8 (C-6), 98.6 (C-8), 78.0 (C-3’’), 76.1 (C-5’’), 74.1 (C-2’’), 70.1 (C-4’’), 61.3 (C-6’’).


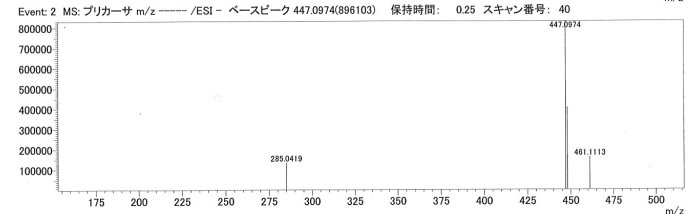
Figure S33: ESI spectrum mass of compound **17**

Figure S34: Full ^1^H NMR spectrum (DMSO-*d_6_*, 600 MHz) of compound **17**

Figure S35: Full ^13^C NMR spectrum (DMSO-*d_6_*, 150 MHz) compound **17**
